# Immune Response in Myocardial Injury: *In Situ* Hybridization and Immunohistochemistry Techniques for SARS-CoV-2 Detection in COVID-19 Autopsies

**DOI:** 10.3389/fmolb.2021.658932

**Published:** 2021-10-26

**Authors:** Pek Yoon Chong, Jabed Iqbal, Joe Yeong, Tar Choon Aw, Kian Sing Chan, Paul Chui

**Affiliations:** ^1^ Department of Pathology, Sengkang General Hospital, Singapore, Singapore; ^2^ Department of Anatomical Pathology, Singapore General Hospital, Singapore, Singapore; ^3^ Institute of Molecular and Cell Biology, A-STAR, Singapore, Singapore; ^4^ Department of Molecular Pathology, Singapore General Hospital, Singapore, Singapore; ^5^ Health Science Authority, Singapore, Singapore

**Keywords:** PCR, COVID-19, autopsy, multiplex, serology

## Abstract

Coronavirus disease-19 (COVID-19) is caused by the newly discovered coronavirus, severe acute respiratory syndrome coronavirus 2 (SARS-CoV-2). While the lung remains the primary target site of COVID-19 injury, damage to myocardium, and other organs also contribute to the morbidity and mortality of this disease. There is also increasing demand to visualize viral components within tissue specimens. Here we discuss the cardiac autopsy findings of 12 intensive care unit (ICU) naïve and PCR-positive COVID-19 cases using a combination of histological, Immunohistochemical/immunofluorescent and molecular techniques. We performed SARS-CoV-2 qRT-PCR on fresh tissue from all cases; RNA-ISH and IHC for SARS-CoV-2 were performed on selected cases using FFPE tissue from heart. Eight of these patients also had positive post-mortem serology for SARS-CoV-2. Histopathologic changes in the coronary vessels and inflammation of the myocardium as well as in the endocardium were documented which support the reports of a cardiac component to the viral infection. As in the pulmonary reports, widespread platelet and fibrin thrombi were also identified in the cardiac tissue. In keeping with vaccine-induced activation of virus-specific CD4^+^ and CD8^+^ T cells, and release of cytokines such as interferon-gamma (IFNγ), we observed similar immune cellular distribution and cytokines in these patients. Immunohistochemical and immunofluorescent localisation for the viral Spike (S-protein) protein and the nucleocapsid protein (NP) were performed; presence of these aggregates may possibly contribute to cardiac ischemia and even remodelling.

## Introduction

SARS-CoV-2 (Covid 19), a novel corona virus was first implicated as the cause of a rapidly spreading infectious upper respiratory illness in late 2019 (Zhu et al., 2020) resulting in an exponential increase in global infections (WHO, 2020). There is much to be done as thenatural history of this disease has yet to be elucidated and whilst there has been an emphasis on pulmonary findings, there are now increasing reports that Covid-19 may also affect the cardiovascular and other organ systems (Babapoor-Farrokhran et al., 2020; Barton et al., 2020). In fact, Yang et al. reported death of a small number of patients who died within a short period of time after admission, in other words, sudden death (Yang et al., 2021). Moreover, sudden cardiac arrest and death had been reported as early as 2020 despite improvement of general condition and constitutional symptoms (Shirazi et al., 2021).

Much about the pathogenesis of SARS-CoV-2 and the heart remains unknown (Siripanthong et al., 2020). Angiotensin (AT) converting enzyme 2 (ACE2), known as the cellular receptor for SARS-CoV-2 is ubiquitously expressed with the highest levels detected in the cardiovascular system (cardiomyocytes, cardiac fibroblasts, vascular smooth muscle cells and endothelial cells) as well as intestine, kidneys and lungs (Grifoni et al., 2020; Le Bert et al., 2020). We discuss the cardiac (and vascular) pathology seen in twelve cases of sudden death in patients who were also Covid-19 positive. We document possible evolution of the disease with little or no medical intervention in a study of these autopsy cases. We also aim to document the cellular immune response observed in the COVID-19 patients.

## Methods

In our series of twelve male autopsy cases, ten cases were sudden unexplained deaths. Case 1 had presented to his physician with anosmia, had a swab taken and was sent home where he was found collapsed the next day before the test results were known. The remaining 9 cases had a variety of complains including chest pain, epigastric pain or discomfort. Case 10 was last heard complaining of chest pain but was found dead 2 days later. Of these only two cases had known premorbid illnesses on record. Case 8 had a history of hypertension whilst case 11, who was unemployed, is the only case with documented premorbid conditions of poorly controlled diabetes and hypertension. He was being managed by his physician for 2 weeks of fever and cough before being tested positive for Covid-19. He collapsed at home a day later.

Two cases were unnatural deaths, having fallen from height (case 2 was admitted to hospital for

observation after 5 days of fever and a positive test, whilst case 9 was admitted in a facility for

well and asymptomatic Covid-19 patients.

All subjects, except for case 11, worked in the construction industry. None of the 12 cases.

Presented with severe respiratory symptoms nor required supplemental oxygen. In cases where clinical history was not available, we have taken the date of the first positive PCR test as the most probable start point of COVID-19 infection and have stratified the patients accordingly.

All twelve cases were referred to Health Sciences Authority for autopsy (mean age = 44.1 year range:27–69 years) (Table 1) under the Second Schedule of the Coroners Act Cap 63A (Revised Edition 2012 Singapore Statutes). All autopsies were carried out either in biosafety level (BL)-BSL3 or BSL4 autopsy facilities. All subjects were male.

**TABLE 1:** Patient characteristics and autopsy findings.

case No.	1	2	3	4	5	6	7	8	9	10	11	12
Gender	M	M	M	M	M	M	M	M	M	M	M	M
AGE	32	46	47	53	30	41	41	48	27	42	69	59
BMI	25.28	19.05	20.62	26.67	16.11	27.1	21.87	18.94	22.41	18.31	21.48	31.7
Ante mortem test (PCR)	YES	YES	NA	NA	NA	YES	NEG	YES	YES	YES	YES	YES
TEST TO DEATH INTERVAL (DAYS)	6 HRS	4 DAYS	NA	NA	NA	42 DAYS	NA	38 DAYS	19 DAYS	47–48 DAYS	1 DAY	41 DAYS
PRIOR MEDICAL CONDITIONS	NIL	NIL	NIL	NIL	NIL	Not known	Not known	Not known	NIL	Not known	DM TYPE 2. HYPT. HYPOTHYROID. OBESE. CKD	Nil
CIRCUMSTANCES OF DEATH	SUDDEN DEATH	Fell from Height	SUDDEN DEATH	SUDDEN DEATH	SUDDEN DEATH	SUDDEN DEATH	SUDDEN DEATH	SUDDEN DEATH	Fell from height	SUDDEN DEATH, DECOMPOSED	ARI; SUDDEN DEATH	SUDDEN DEATH
HEART WT (gm)	345	235	419	410	245	404	245	340	222	244	463	398
HEART GENERAL DESCRIPTION	SOFT AND FLABBY.	SOFT AND FLABBY.	SOFT AND FLABBY	TRANSMURAL RUPTURE, ANTERIOR-ANTEROSEPTAL WALL, JUNCTION OF UPPER 2/3 AND DISTAL 1/3 LV.	SOFT AND FLABBY	SOFT AND FLABBY, FIBRINOUS ADHESIONS OVER RA, RV ENDOCARDIUM	GROSSLY NORMAL	GROSSLY NORMAL	GROSSLY NORMAL	DECOMPOSED	HEART ENLARGED	HEART ENLARGED
CORONARY arteries	LCA: PINPOINT RESIDUAL LUMEN	NORMAL.	LCA ATHEROSCLEROTIC WITH 50% OCCLUSION OF LAD.	LAD 10–25%. DARK RED THROMBUS, TOTAL OCCLUSION	LAD- ORGANISING THROMBUS.	MILD ATHEROSCLEROSIS						
LAD 10–25%	PROX LAD 75%-	LCA 25/LAD 90/LCX 75/RCA PINPOINT	NAD	LAD 10–25	LCA 10 LAD 90-FIRST DIAGONAL PINPOINT	LCA 50 LAD25						
SEROLOGY (IgM + IgG)	CLOTTED	CLOTTED	POSITIVE; COI 2.22	POSITIVE; COI 2.38	CLOTTED	POSITIVE; COI 89.8	POSITIVE; COI 9.91	POSITIVE; COI 114	POSITIVE; COI21.5	UNSUITABLE FOR ANALYSIS	NO; COI 0.534	POSITIVE; COI:8.73
Swab (Nasal)	DETECTED	DETECTED	PRESUMPTIVE POSITIVE	NOT DETECTED	DETECTED	NOT DETECTED	NEGATIVE	NEGATIVE	NEGATIVE	DETECTED	DETECTED	NEGATIVE
Swab (PNS)	DETECTED	DETECTED	DETECTED	DETECTED	PRESUMPTIVE POSITIVE	NOT DETECTED	DETECTED	DETECTED	DETECTED	DETECTED	DETECTED	NEGATIVE
Swab (Tracheal)	DETECTED	DETECTED	PRESUMPTIVE POSITIVE	NOT DETECTED	DETECTED	NOT DETECTED	NEGATIVE	NEGATIVE	DETECTED	NEGATIVE	DETECTED	NEGATIVE
Swab (Ileal)	DETECTED	DETECTED	NOT DETECTED	INCONCLUSIVE	DETECTED	DETECTED	NEGATIVE	NEGATIVE	NEGATIVE	DETECTED. STRONG POSITIVE	PRESUMPTIVE POSITIVE	NEGATIVE
Swab (CNS)	DETECTED	DETECTED	NOT DETECTED	NOT DETECTED	NOT DETECTED	NOT DETECTED	NEGATIVE	NEGATIVE	NEGATIVE	NEGATIVE	DETECTED	NEGATIVE

LCA; Left coronary artery, LAD: Left anterior descending artery, PROX LAD: Proximal Left anterior descending artery, LCX: Left circumflex artery, RCA: right coronary artery.

## Results

### Vasculature

**FIGURE 1:**
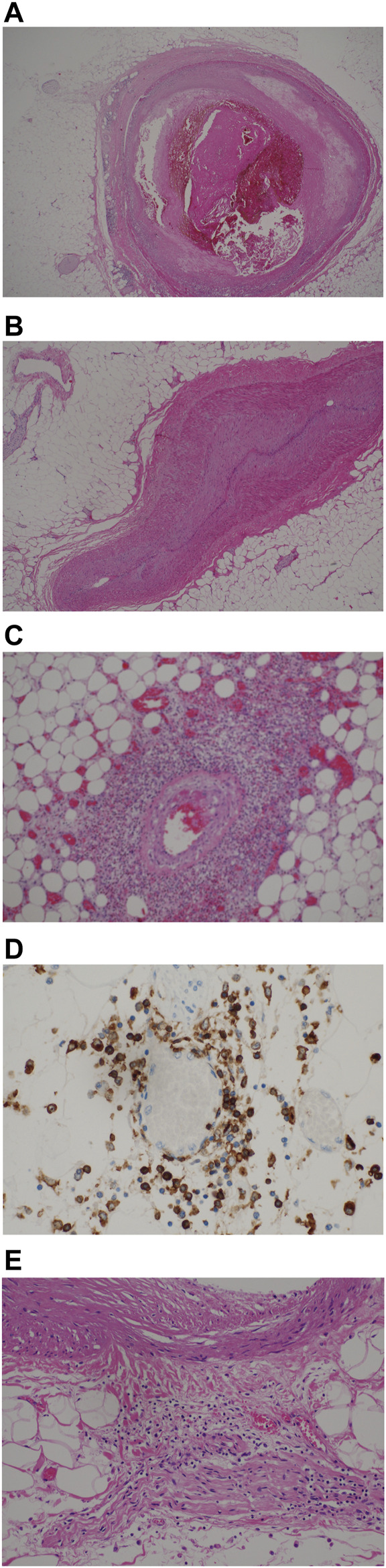
**(A)** Coronary vessel with fresh fibrin thrombus, moderate inflammation, mild fibrosis (H&E, x200). **(B)** apposition of vascular endothelium (H&E, x200). **(C)** Perivascular inflammation around epicardial vessel (H&E, x200). **(D)** CD4-positive T-cells in perivascular tissue, 200x. **(E)** Perivascular inflammation extending to adipose tissue and involving peripheral nerve (H&E, x200).

The findings are stratified with respect to known or estimated duration of symptoms. The earlier group comprised 5 cases with symptom duration ranging between <12 h and 5 days. In 4 of these, we observed complete occlusion of the coronary artery by fresh fibrin thrombi; the epicardial coronary vessels showed pre-existing mild atherosclerosis (Figure 1A). In a fifth patient (case 2), we observed that the patency of the arterial lumen was compromised by apposition of the endothelium (Figure 1B).

An inflammatory infiltrate was seen around epicardial vessels of varying diameter, predominantly in a perivascular location, extending into the outer layers of the vessel wall and outwards into the pericardial adipose tissue (Figure 1C, patient 3, Table 2). On cross sections, the interface of the more intense inflammatory infiltrates imbued a stellate appearance. The inflammatory cells were predominantly lymphoid in nature, particularly CD4-positive (IHC) with scanty CD8-positive T-cells and CD20-positive B cells (Figure 1D). In some cases, the latter two subtypes of lymphocytes were virtually absent. Except for two cases where the eosinophilic infiltrate was heavy, eosinophils and monocytes were also noted in smaller amounts. Involvement of nerves by chronic inflammation were also seen Figure 1E.

**TABLE 2:** Microscopic findings; key: Light yellow <7 days; Pink >7 days.

case no	1	2	3	4	5	7	11	9	6	8	10	12
Interval from ante-mortem PCR to autopsy	6 h	4 days	NA	NA	NA	NA	1 day	19	42 days	38 days	47–48 days	41 days
Epicardial vessel lumen	occluded	occluded (lumen closed by apposition)	occluded	occluded	occluded	80%	occluded atherosclerotic with inflammatory infiltrates	myointimal thickening	myointimal thickening	myointimal thickening	myointimal thickening	myointimal thickening
Fresh thrombus	yes	no	yes	yes	yes	no	no	no	no	no	no	no
Fibrin thrombi	yes	yes	yes	yes	yes	Yes	yes	yes	Yes	yes	Autolytic changes	yes
Perivascular inflammation	Yes 3 + mixed inflammatory cells	yes 1 + lymphocytic infiltration	yes 2 + lymphocytic infiltration	Yes 1 + lymphocytic infiltration	Yes 2 + mixed inflammatory cells	yes	Lymphoid aggregates	1 + lymphocytic infiltration	1 + lymphocytic infiltration	Lymphoid aggregates	Autolytic changes	Lymphoid aggregates
Myocardium												
inflammatory infiltrate	2 + lymphocytic	no	3 + lymphocytes and eosinophils	1 + Lymphocytes	3 + lymphocytes and eosinophils	2 + lymphocytes	scanty lymphocytes	1 + LYMPHOCYTES	2 + lymphocytes	1 + lymphocytes	Autolytic changes	1 + lymphocytes, eosinophils

**FIGURE 2:**
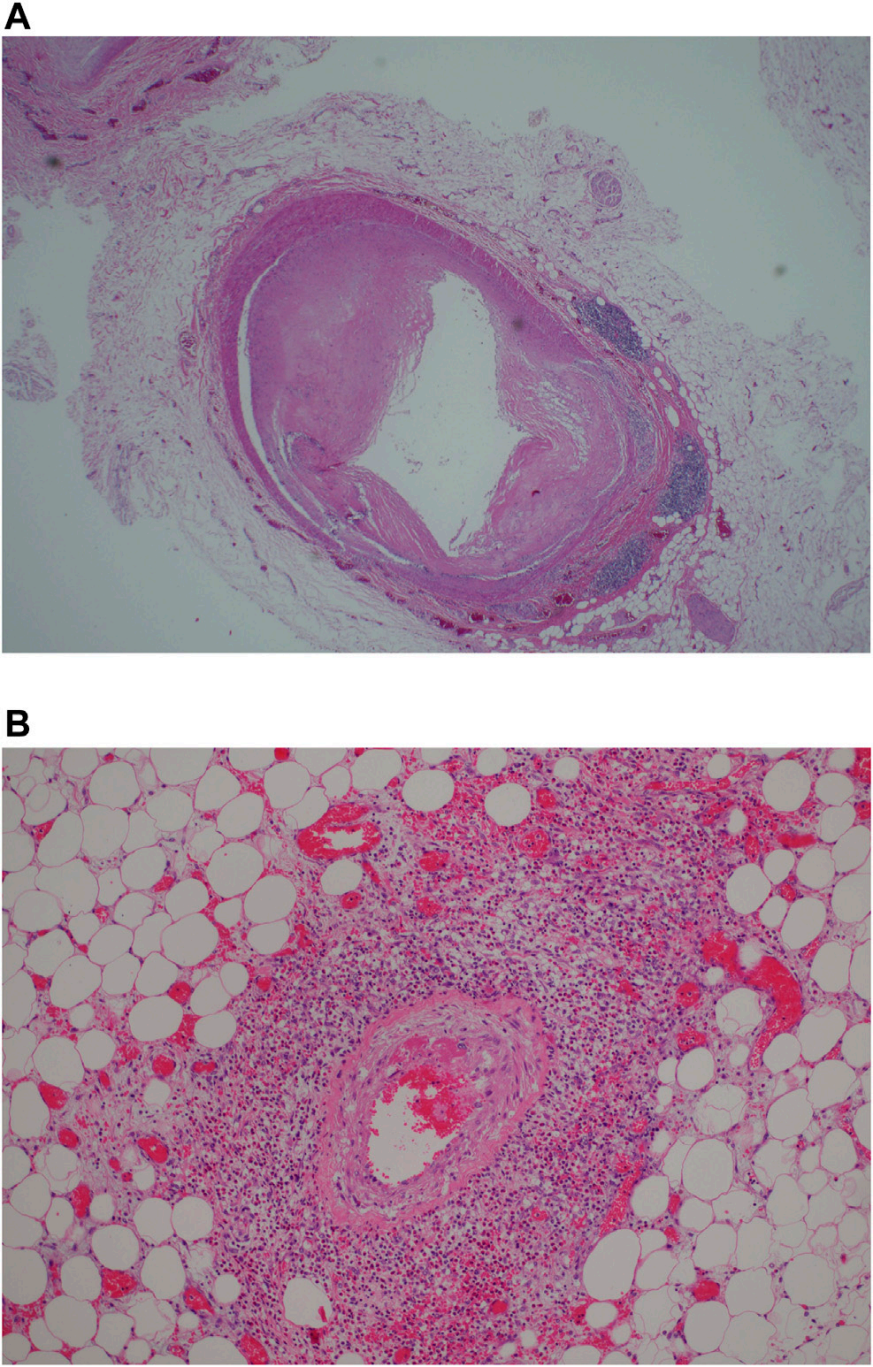
**(A)** Remodeling of myocardial vesels showing myointimal hypertrophy/thickening with perivascular lymphoid aggregates (H&E, x200). **(B)** Perivascular inflammation involving epicardial vessel (H&E, x200).

In cases where symptoms persisted for more than 14 days, the inflammatory infiltrate was less prominent. The coronary vessels showed medial and intimal hypertrophy with focal dilatation, outpouching or tortuosity of vessels giving an irregular appearance (Figure 2A, case 5 A fine perivascular fibrosis was seen around both thin and thick-walled vessels of varying diameters including capillary vessels. The pericardial adipose tissue was involved in the inflammation in all cases, irrespective of duration of symptoms (Figure 2B).

### Myocardium

**FIGURE 3:**
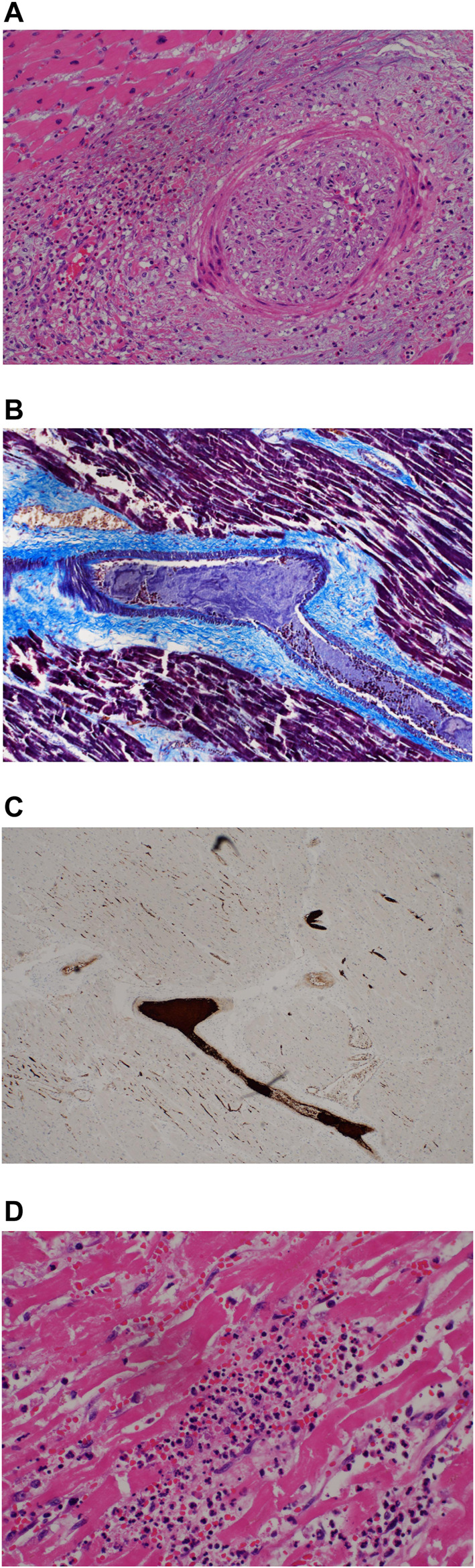
**(A)** Occlusion of coronary artery lumen by proliferation of spindled cells (H&E, x200). **(B)** Fibrin thrombi. MSB stain (200x). **(C)** Platelet thrombi, CD61 immunohistochemical stain (200x).

The perivascular inflammatory infiltrate described previously was observed to follow the vessels into the myocardium and could be seen extending along the longitudinal axis of the vessels. In some cases, the inflammatory infiltrates were seen around the perivascular spaces, within the wall and lumen. Again, the lymphoid cells were predominantly of CD4 lineage with virtually no CD8-positive T-cells or CD20-positive B-cells. Obliteration of the vascular lumen by a proliferation of spindled myofibroblasts was seen in one case (Figure 3A, case 5).

Extensive fibrin and platelet thrombi were noted in the myocardial vessels as well as in the myocardial microvasculature. Mature fibrin thrombi were observed with MSB stains (Figure 3B); intravascular platelet aggregates were demonstrated by CD61 antibody (Figure 3C).

**FIGURE 4:**
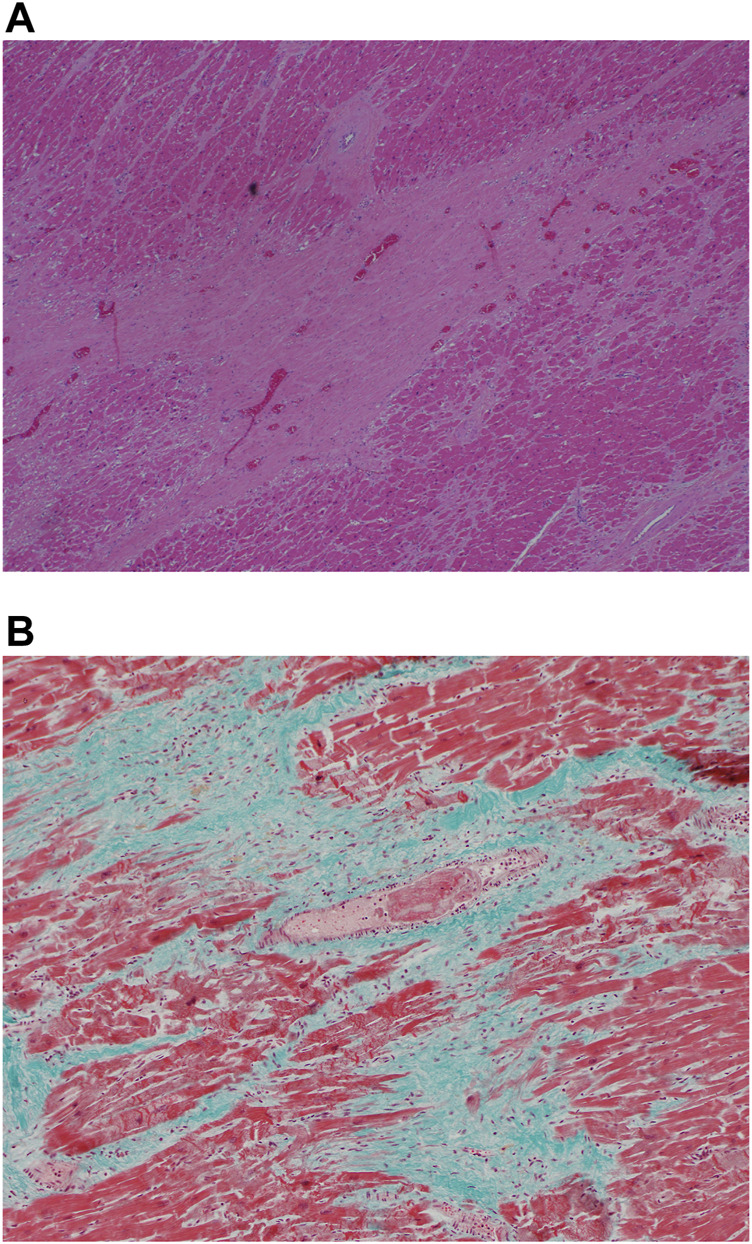
**(A)** Myocarditis with myocyte necrosis (H&E, x200). **(B)** Stellate myocardial fibrosis (H&E, 200x). **(C)** Masson trichrome stain confirming fibrosis (200x).

Myocarditis as evidenced by chronic inflammation and myocyte necrosiswas marked in 4 of the 5 earlier cases but could be seen in patchy fashion even in the later cases (Figure 4A, case 3) The inflammatory cells comprised a mixed population of lymphoid cells, neutrophils, eosinophils and monocytes; eosinophils were prominent in case 3,. Granulation tissue reaction with presence of reactive stromal cells was demonstrated in 2 of the earlier cases whilst a patchy stellate myocardial fibrosis was noted in the later cases (more than 7 days) (Figure 4B,C, case 8). This fibrosis could also be seen sweeping along the long axis of the myocardial vessels appearing to mirror the inflammatory infiltrate in the earlier cases. Within the fibrous tissue, thin-walled vessels were noted. It is unclear if these vessels represent residual vasculature or neovascularization. (Figure 4D, case 8).

### Endocardium

**FIGURE 5:**
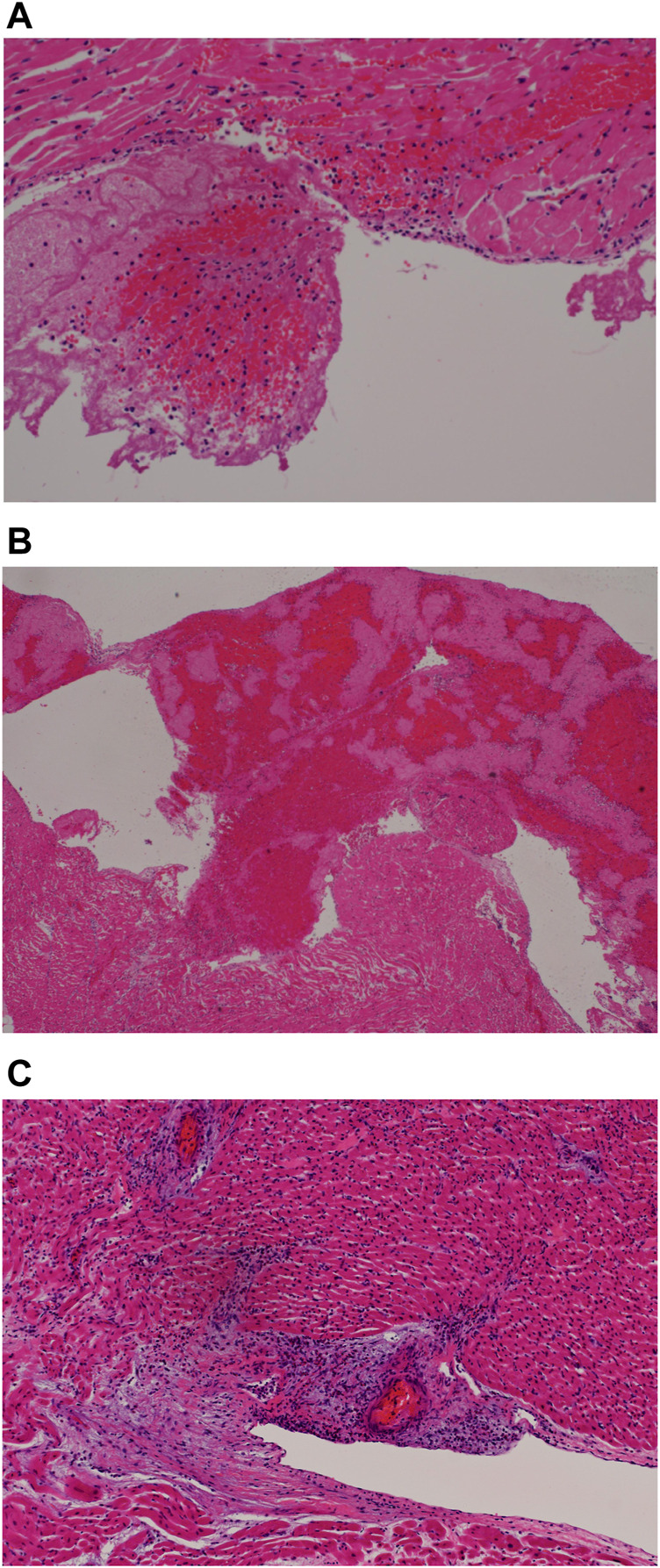
**(A)** Endocardial fresh blood clot (H&E, 200x). **(B)** Endocardial organizing thrombus (H&E, 200x). **(C)** Endocarditis (H&E, 200x).

Focal infiltration of the endocardial lining by mononuclear cells was noted in the earlier cases (Figure 5A–C, case 5).

### RNAscope in-Situ Hybridization

**FIGURE 6:**
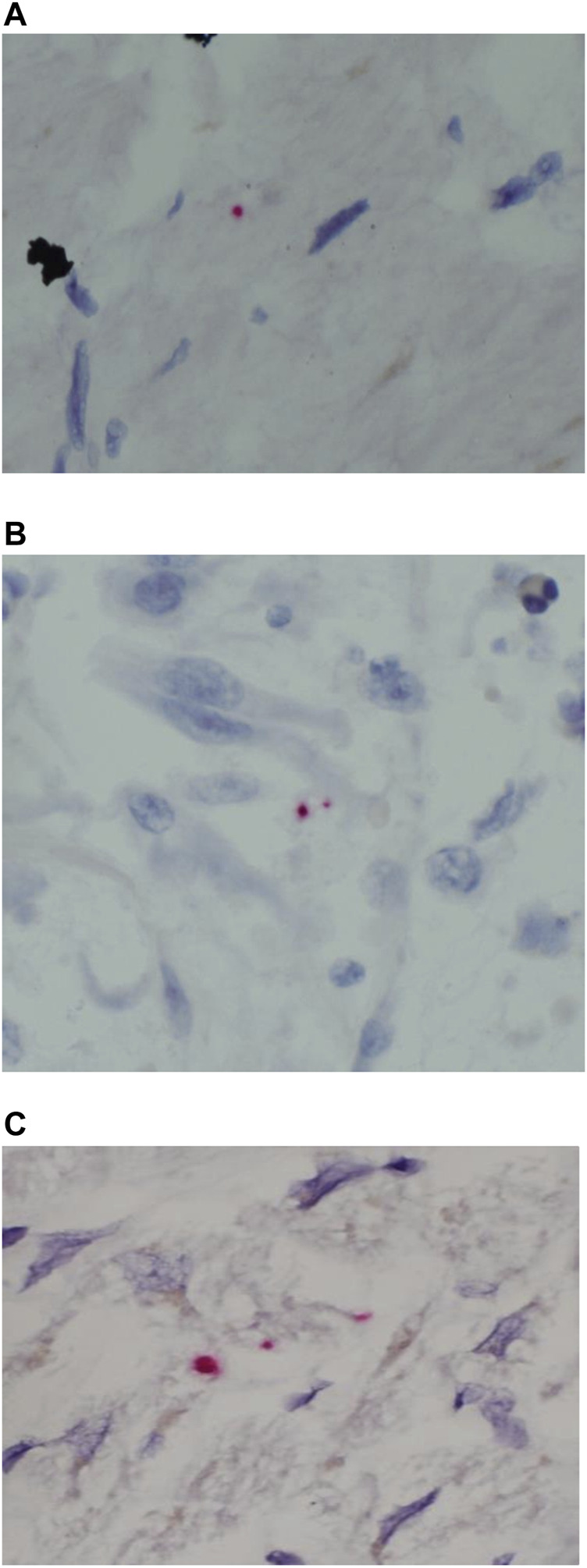
SARS-CoV-2 ISH in myocardium from the autopsy of patients who died secondary to COVID-19 infection. **(A–C)** Positive reactions for the probes directed against SARS-CoV-2 S-protein (red dot) original magnifications ×400).

RNAscope assay following the manufacturer’s protocols was applied to five cases. Weak positive signals were detected within myocytes in three cases (cases 2,3 and 5). Co-localization of SARS-CoV-2 with its entry receptor ACE2 and serine protease TMPRSS2 (type II transmembrane serine protease) in different cell types, using RNAscope *in situ* hybridization, was found in various compartments of the heart, such as endothelial smooth muscle, myocardium, fibroblastic and inflammatory cells (Figure 6).

### Immunohistochemistry

**FIGURE 7:**
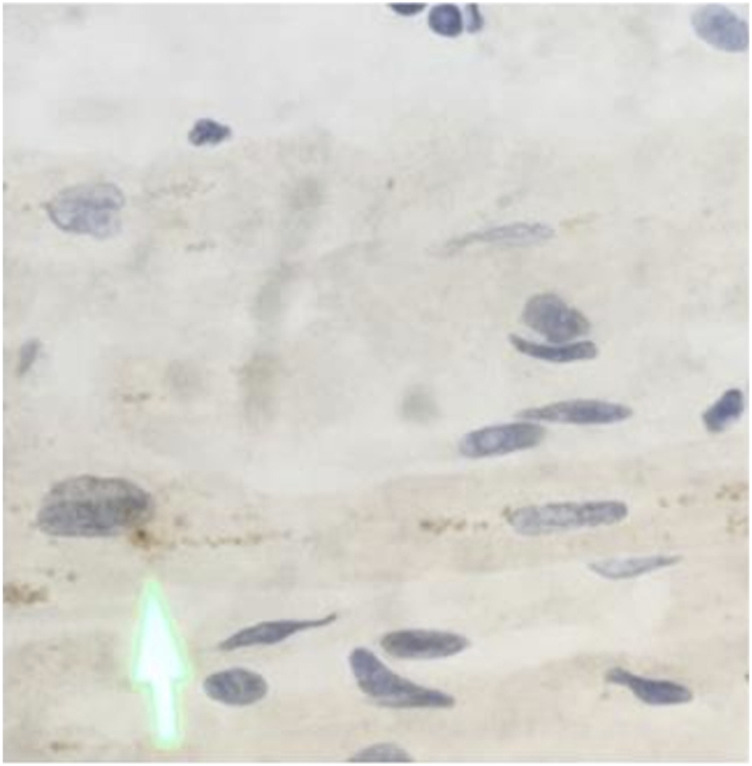
Immunohistochemical analysis in myocardium from the autopsy of patient who died secondary to COVID-19 infection **(A)** SARS-CoV-2 is positive (arrow) in the myocardial tissue, original magnifications ×400.

Detection of viral NP in myocardium was attempted using immunohistochemistry and identified in two cases including one case with coexisting myocardial viral S protein on RNAscope assay. The second case was 38 days post-covid-19 infection and viral NP-protein was detected within the vascular lumen as well as the immediate perivascular space (Figure 7, case 8).

### mIHC/IF

**FIGURE 8:**
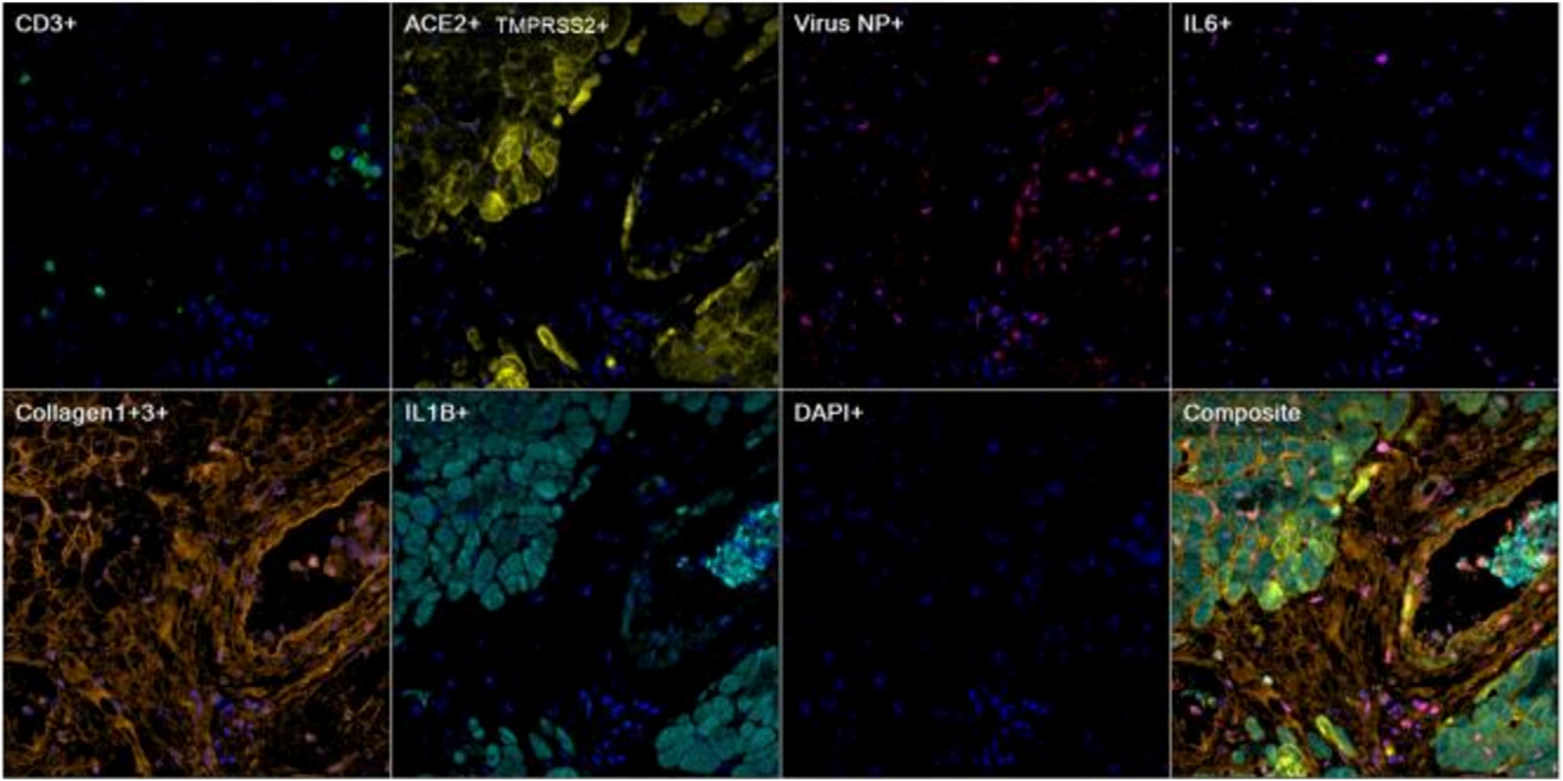
Representative images of heart tissue stained using multiplex immunohistochemistry/immunofluorescence (mIHC/IF) [CD3 (green), ACE2/TMPRSS2 (yellow), Virus NP (red), IL6 (magenta), Collagen1+3 (orange), IL1B (cyan), DAPI (blue)] (Magnification, 400X).

We investigated the presence of the SARS-COV2 NP protein (Shirazi et al., 2021; Siripanthong et al., 2020; Le Bert et al., 2020; Grifoni et al., 2020) and the associated immune microenvironment by using multiplex IHC/IF technique (Ni et al., 2020; Thieme et al., 2020; Stack et al., 2014; Abel et al., 2014; Lovisa et al., 2015; Garnelo et al., 2015; Yeong et al., 2017; Garnelo et al., 2017; Esbona et al., 2016; Mlecnik et al., 2016; Nghiem et al., 2016; Feng et al., 2016; Lim et al., 2018). The SARS-COV2 NP protein was found predominantly near the perivascular regions colocalizing with receptors of SARS-COV2, ie ACE2 and TMPRSS2 (Yeong et al., 2019; Lam et al., 2019) (Figure 3). Interestingly, the pathogenic cytokines such as IL-6 (Xu et al., 2020a; Hoffmann et al., 2020; Qin et al., 2020; Wang et al., 2020) and IL-1β (Huang et al., 2020a; Xu et al., 2020a; Diao et al., 2020; Shi et al., 2020) were also detected in close proximity in the background of fibrosis highlighted by the expression of Collagen I and III (Liao et al., 2020; Wen et al., 2020).. (Figure 8)

**FIGURE 9:**
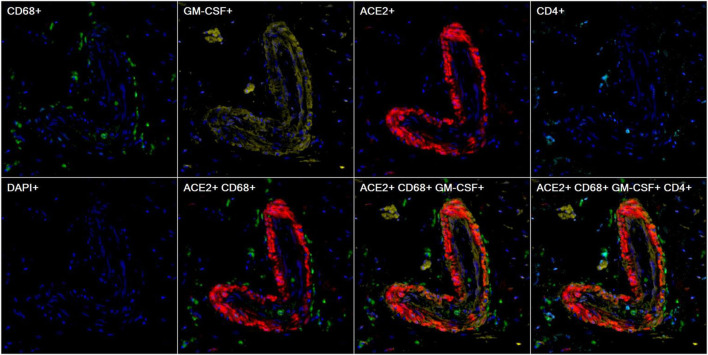
Representative images of heart tissue stained using multiplex immunohistochemistry/immunofluorescence (mIHC/IF) [CD68 (green), GM-CSF (yellow), ACE2 (red), CD4 (cyan), DAPI (blue)] (Magnification, 200X).

We further demonstrated that some of the ACE2^+^ cells were macrophages which are in line with previous reports (Delpino and Quarleri, 2020; George et al., 2020). Colocalization of the pathogenic cytokine GM-CSF (Diao et al., 2020) as well as surrounding T-cells were also observed in the proximity (Figure 9, case 8).

**FIGURE 10:**
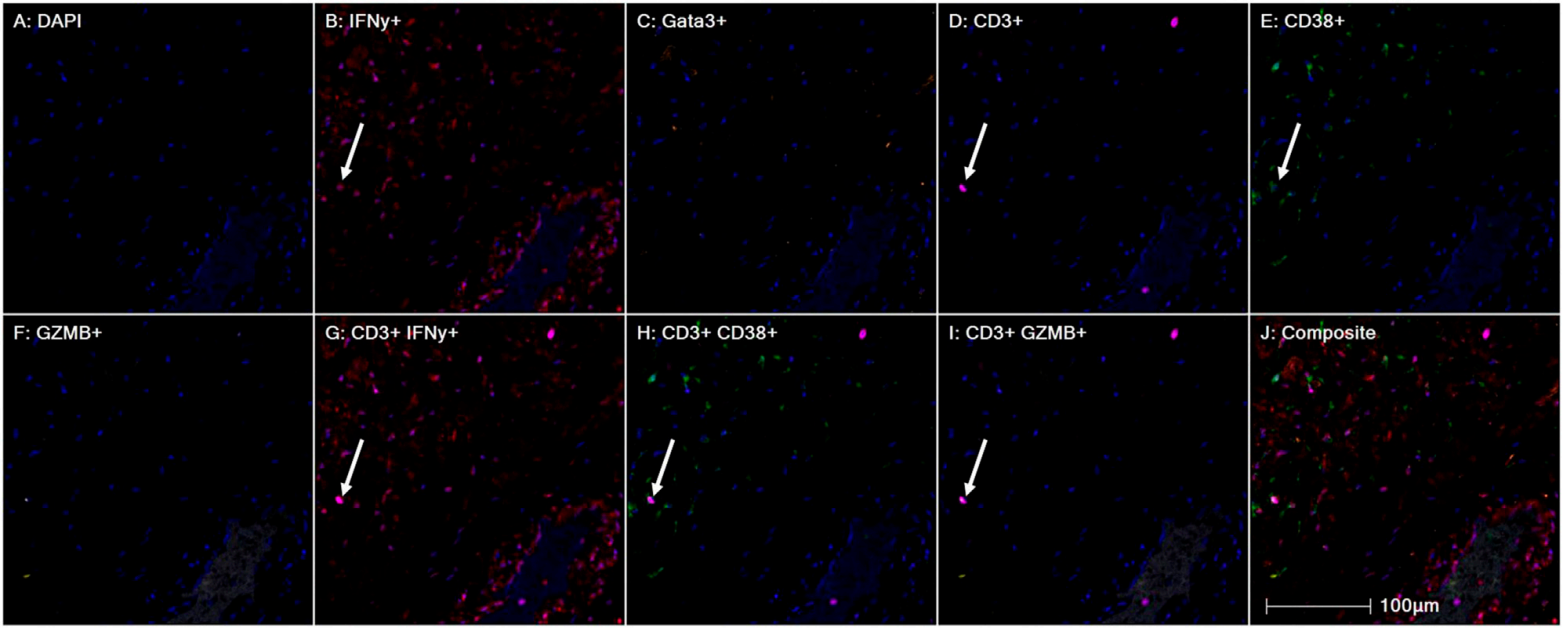
Representative images of heart tissue stained using multiplex immunohistochemistry/immunofluorescence (mIHC/IF) Interferon gamma (red), GATA3 (orange), CD3 (magenta), CD38 (green), Granzyme B (yellow) (Magnification, 200X).

Furthermore, we interrogated the immune phenotype of the T-cells present in the peri-vascular regions whereby Th2 cells, highlighted by absence of GATA3 nuclear stain, were virtually absent (Robinson et al., 2020). However, few T-cells demonstrated a Th-1 cell-like phenotype which expressed pathogenic cytokines such as interferon-gamma (Zheng and Flavell, 1997; Xu et al., 2020a; Diao et al., 2020; Weiskopf et al., 2020; Yao et al., 2020). Some of the T-cells expressed granzme-B signifying the effector function of cytotoxic T cells (Thevarajan et al., 2020; WHO, 2020; Zheng et al., 2020), as well as CD38 which has been widely reported as one of the SARS-COV2-specific T-cell marker (Huang et al., 2020a; Xu et al., 2020b; Craver et al., 2020; Thevarajan et al., 2020; Wang et al., 2020) (Figure 10, case 8).

### SARS-CoV-Ab Serology

COI values for 7 of the 8 patients were clearly positive ranging from 2.22 to 114; it was 0.534 in the remaining patient. The range in 362 prepandemic male (age: mean, SD - 43.3, 14.5 years) sera on this assay was 0.066–0.373 (data not shown).

## Discussion

We document 12 autopsy cases with cardiac changes. Four of these cases had histologic evidence of myocarditis with marked predominantly lymphocytic and some eosinophilic infiltrates. The presence of SARS NP protein in a perivascular location, the proximity of T-cells and cytokine GM-CSF bum IHC/IF appears to corroborate changes observed at the light miscroscopy. Whether these changes were purely due to an underlying ischemic heart disease or whether the process was exacerbated by the viral infection is unclear.

Myocardial cells are a potential target of SARS-CoV-2, and myocarditis has been reported in a limited series in China, where 7% of deaths were attributed to myocardial damage with circulatory failure without a clear, definite diagnosis of myocarditis (Cheung et al., 2020). Others have described fulminant myocarditis in the setting of high viral load, with autopsy findings of myocardial inflammatory infiltrate, but without evidence of myocardial COVID-19 disease (Robinson et al., 2020). Although much of the published literature has focussed on the pulmonary changes, findings from our autopsy series demonstrate that significant cardiac pathology may be associated with COVID-19 infection. It has already been postulated that in addition to coronary plaque destabilization and hypoxia, the possible mechanisms of COVID-19-related myocardial injury could be direct damage to the cardiomyocytes, chronic inflammation, myocardial interstitial fibrosis, interferon-mediated immune response and exaggerated cytokine response by T-cells (Babapoor-Farrokhran et al., 2020).

The records of the Forensic Department, Singapore show that in 2018, a total of 1,414 cases were autopsied and 18 were certified as myocarditis. In 2019 there were 1,262 cases of which 23 were certified as myocarditis. 131 cases were certified as Coronary heart disease in 2018 and 108 in 2019. A small autopsy series of four non-COVID-19 deceased patients during the same period did not reveal any significant myocardial inflammation (Supplementary Table S2).

In SARS autopsies (Chong et al., 2004) we reported pulmonary thromboembolic, deep vein thrombosis, and marantic valvular vegetations with widespread intravascular fibrin thrombi. As the SARS patients had a history of admission to intensive care units (ICU), it could be argued that these changes were related to ICU support and therapy. However, our current cases are all ICU-naïve.

SARS-CoV-2 is known to use the ACE2 receptor as a channel for entrance into the cell as receptors have been reported to be present in cardiac smooth muscle, vascular smooth muscle and endothelial cells as well as pneumocytes and enterocytes (Dandekar and Perlman, 2005; Yajima and Knowlton, 2009). It is possible that the virus enters these cells including the cardiac myocytes and that the immediate innate cytokine response may cause the initial myocardial damage early in the infection and that the arrival of the T-lymphocytes would further intensify this. Our studies have been able to demonstrate viral signals within the cardiac myocytes using both ISH and mIF assays. The latter has also demonstrated increased cytokine and interferon activity within the myocardium in perivascular locations co-locating with SARS-CoV-2specific T cells as well as macrophages. We note that in staining for the ACE2 receptor, the distribution of these receptors is not uniform throughout the cardiac and vascular samples. This underlying variation may underscore the heterogenous distribution of cardiac injury seen in our cases.

Yajima and Knowlton (2009); Wick et al. (2004) Immunohistochemical findings in our cases suggest the presence of virus within the vessel lumen and wall as well as within immediate perivascular space together with the presence of activated lymphocytes and cytokine activity (Figure 4). Macrophage/monocytes are known to be able to transport Corona viruses and studies have also suggested that T-lymphocytes may be similarly infected (Wick et al., 2004; Yajima and Knowlton, 2009). Hence whether this vascular inflammation is a direct reaction to the existence of the virus within the endothelium or a combination of dysregulated T cell, cytokine and humoral response remains to be seen. Consequently, it is tempting to propose that the observed perivascular and intravascular inflammation modulates the vascular integrity leading to vascular remodelling in the long-term.

The cytokine and inflammatory cell activity within the lumen are interesting as this suggests injury to the endothelial lining. Our cases show widespread platelet and fibrin thrombi within myocardial vessels and the myocardial microvasculature which appears to persist even in cases with a prolonged disease. The presence of possible NP protein signals within the lumen of the vessel may also suggest persistence of viral presence in circulating monocytes as another stimulus for the microembolic phenomena (Figure 4).

One of our cases also showed marked fibroblastic activity within intramyocardial vessels, suggesting possibly immune-mediated injury to endothelial lining possibly contributing to a stenosing lesion (Thieme et al., 2020). In recent years, pro-fibrotic role of the innate immune system has become apparent. Early events of fibrosis comprise inflammatory changes (Wick et al., 2010), including recruitment of mononuclear inflammatory infiltrates. Although, the initial events in activation of host defence mechanisms are still largely unknown, It has been proposed that viral myocarditis may have several phases from predisposition or susceptibility of the cardiac myocyte to infection, entry and active viral replication in the myocyte, persistence of the viral genome without detectable replication and remodelling without detectable viral genome (Varga et al., 2020).

Using mIF methods, we have demonstrated presence of IL-1B and IL6 activity within collagen fibers in the regions of myocardial fibrosis, both of which have been known to play a profibrotic role (Huang et al., 2020a; Patil et al., 2020). It is possible that profibrotic cytokines and mediators released during myocarditis phase in susceptible individuals activate fibroblasts and stimulate fibroblast differentiation leading to subsequent cardiac remodelling. One further interesting point is the persistent epicardial adipose tissue injury seen in all cases manifested by small aggregates of CD4^+^ T-lymphocytes around small thin-walled vessels and edematous tissue in the early cases to patchy aggregates of lymphocytes and contraction of the epicardial layer. As this epicardial adipose tissue has been postulated to be active in secretion of endocrine and paracrine substances, this persistent inflammation at this location may further compound vascular injury (Huang et al., 2020b).

Interestingly, use for SARS-CoV2 antibody titer has not been reported before for forensic purposes. The Roche assay detects total antibodies to the nucleocapsid protein. The results are reported as a cut-off index (COI); COI >1.4 are considered positive. This suggests that the COI of 0.534 may represent a subclinical antibody titer. Our findings suggest a possible forensic application for the Roche serology test.

Although direct SARS‐CoV‐2-induced myocardial is a consideration, COVID‐19‐associated cardiac damage is widely attributed to exaggerated immune response. One of the early reports describing myocardial inflammation in SARS‐CoV‐2 infection reported fulminant myocarditis with elevated IL‐6 levels along with other cardiac injury markers (Wong et al., 2004). Various cohort‐based studies also showed an increased cytokine production during COVID‐19 infection, and cytokine storm in these patients was found to be associated with the disease severity and patient survival (Molenkamp et al., 2020). The immune response in SARS patients is mainly mediated through the Th1‐cell activity as opposed to SARS‐CoV‐2 infection, where an imbalance between both Th1 and Th2 activity was found to support the inflammatory surge (Peiris et al., 2003; Corman et al., 2021). Overall, evidence from the published studies and ours implies that the SARS‐CoV‐2‐induced inflammatory response may be a possible cause of cardiac damage in patients and could be targeted for therapeutic interventions. The robust elicitation of IFNγ-producing CD4^+^ T cells in our studies indicates that a cellular immune response with potential anti-viral properties mirrors the strong neutralizing antibody and cytokine response seen in vaccine trials (Sahin et al., 2020). More recently, Bearse *et al* demonstrated that cardiac infection with SARS-CoV-2 was common among patients succumbing to COVID-19 infection. This study also showed that SARS-CoV-2-associated cardiac infection was associated with more cardiac inflammation and electrocardiographic changes (Bearse et al., 2021). Nonbiologic immunosuppression is associated with lower incidences of myocarditis and cardiac infection by SARS-CoV-2. In our series none of the patients received COVID-19 specific treatment.

Decrease of eosinophils was a critical event described in sudden deaths, which is consistent with previous report that eosinophils may predict the outcome of COVID-19 progression (Babapoor-Farrokhran et al., 2020). Patients with high percentage of neutrophils or neutrophils count had an increased risk of sudden death, probably due to cytokine storm activated by neutrophils (Wu et al., 2020). Unfortunately we did not have corroborating evidence to monitor immune cell counts or inflammatory biomarkers.

Exposure and susceptibility to COVID-19 are partly influenced by occupation and working environment. Migrant workers as in our cohort constituted a significant proportion of the workforce in sectors that have remained active throughout the crisis, such as construction work, logistics and deliveries. Several confounding factors such as inability to work in isolation, lack of access to private transportation, close physical proximity with coworkers and in some instances lack of adequate protective equipment render these workers particularly susceptible.

Although our study only examined male patients, the observed myocarditis is still concordant with other studies. In one study, the overall risk of patients with COVID-19 was nearly 16 times the risk for myocarditis compared with patients who did not have COVID-19. Patients with myocarditis were more commonly male (59.3 versus 41.7%). Despite the limitations, the observed myocardial lesions in our cohort may still be concordant with other studies (Boehmer et al., 2021).

Despite limited existing evidence, our study may provide relevant clues to associate sudden death of COVID-19 patients and potential risk factors. However, several limitations should be considered in our study: 1) it was a retrospective, single-center study and we were not able to conduct all radiographic or laboratory examinations in our subjects 2) interpretation of our findings might be limited by the small sample size 3) data collected for each patient may not be uniform as they represented different disease stages, which might lead to bias in clinical characteristics. Finally, as this is a descriptive/observational study, further mechanistic explanation needs to be clarified. Despite these limitations, our study demonstrated some insights into the characteristics of COVID-19 patients potentially at risk of sudden death. This would help physicians to effectively triage patients with particularly poor prognosis on admission to reduce the fatality rate.

## Conclusion

Understanding the pathogenesis of COVID-19 infection is vital to the proper management of this disease. Conventional autopsy studies combined with state-of-the art molecular techniques are an integral part of this process. Here we highlight the incidence of increased cardiac and vascular events in COVID-19-infection which may underlie inflammatory syndromes (Wong et al., 2004) and raise the possibility for long term complications of potentially cardiotropic and persistent virus. Either persistent viraemia or migration of infected immune cells from the extracardiac locations likely occurs in COVID‐19 patients which may exacerbate underlying ischemic myocardial injury. The association of COVID19 NP protein with endothelial cells, cardiac smooth muscle cells warrants further investigation particularly in COVID19 recovering patients. The possible contribution to susceptibility of cardiac complications by gender, nutrition, genetics or viral mutation should also be considered.

## Methodology

### Real-Time Polymerase Chain Reaction

The inoculated swabs were tested at Singapore General Hospital Molecular Laboratory either using the automated Roche Cobas 6,800 System (Roche Molecular Systems, Inc., Branchburg, NJ) cobas SARS CoV-2 test, a dual-target (E gene and ORF1) qualitative real-time RT-PCR assay, or using our in-house developed SARS CoV-2 RT-PCR assay targeting the SARS-CoV-2 E-gene region (modified from the protocol published by Corman et al. (2021).

### Multiplex Immunohistochemistry/Immunofluorescence

mIHC/IF was performed using an Opal Multiplex fIHC kit (Akoya Bioscience, Menlo Park, California, USA), as previously described by our group and in other studies (Abel et al., 2014; Stack et al., 2014; Garnelo et al., 2015; Lovisa et al., 2015; Esbona et al., 2016; Garnelo et al., 2017; Yeong et al., 2017; Grifoni et al., 2020; Le Bert et al., 2020; Ni et al., 2020; Siripanthong et al., 2020; Thieme et al., 2020; Shirazi et al., 2021). Slides were labelled with primary antibodies, followed by appropriate secondary antibodies (see Supplementary Table S1). Particularly for this panel, we followed the detailed protocol that our group previous reported as protocol manuscript (Nghiem et al., 2016) and hereby briefly described.

FFPE tissue sections were cut onto Bond Plus slides (Leica Biosystems Richmond) and heated at 60°C for 20 min (Feng et al., 2016). Tissue slides were then subjected to deparaffinisation, rehydration and heat-induced epitope retrieval (HIER) using a Leica Bond Max autostainer (Leica Biosystems Melbourne), prior to endogenous peroxidase blocking (Leica Biosystems Newcastle). Slides were incubated with primary antibodies followed by application of polymeric HRP-conjugated secondary antibodies (Leica Biosystems Newcastle). An appropriate Opal fluorophore-conjugated Tyramide signal amplification (TSA) (Akoya Bioscience, Menlo Park, California, United States) was then added at 1:100 dilution. Slides were rinsed with washing buffer after each step. Following TSA deposition, slides were again subjected to HIER to strip the tissue-bound primary/secondary antibody complexes and ready for labelling of the next marker. These steps were repeated until all six markers were labelled and finally added with spectral DAPI (Akoya Bioscience, Menlo Park, California, United States) at 1:10 dilution. Slides were mounted in ProLong Diamond Anti-fade Mountant (Molecular Probes, Life Technologies, United States) and cured in the dark at room temperature for 24 h. Images (viable tumour regions were selected by pathologists) were acquired for each case using a Vectra three pathology imaging system microscope (Akoya Bioscience, Menlo Park, California, United States) then analysed and scored by pathologist with inForm software (version 2.4.2; Akoya Bioscience, Menlo Park, California, United States) (Zheng and Flavell, 1997; Weiskopf et al., 2020) as well as HALO TM (Indica Labs) (Xu et al., 2020a; Hoffmann et al., 2020).

### RNA in-Situ Hybridization

For RNAscope RNA-ISH (Advanced Cell Diagnostics) analysis of EBNA1, standard RNAscope manufacturer’s protocols were followed using the RNAscope H2O2 and protease pretreatment kit (ACD, reference# 322,381), RNAscope Target retrieval buffer (ACD, reference# 322,000), and appropriate positive and negative RNA probes for controls.

### Immunohistochemistry

IHC was performed on the FFPE tissue samples as previously described (Mlecnik et al., 2016; Qin et al., 2020; Wang et al., 2020). Tissue sections (4 μm thick) were labelled with antibodies targeting SARS-CoV-2 NP, as listed in Supplementary Table S1. Appropriate controls were included. To evaluate the antibody-labelled sections, images were captured using an IntelliSite Ultra-Fast Scanner (Philips, Eindhoven, Netherlands).

### SARS-CoV-Ab Serology

Post-mortem arterial blood from all 8 COVID19 RT-PCR positive patients was tested SARS-CoV-Ab was measured on the Cobas e801 immunoassay analyzer (Roche). This assay measures total antibodies directed against the nucleocapsid protein. The assay has a specificity of 99.9% (714/715) and a sensitivity of 48.2% within the first week after positive RT-PCR results (n = 189) rising to 97.1% 14 days post-PCR diagnosis (n = 70). COI>1.0 are considered positive. for SARS-CoV-2 antibodies on the Roche Cobas e801 analyzer as per the manufacturer’s instructions. The performance of this Roche assay has been evaluated and verified recently (Lau et al., 2020).

## Data Availability

The raw data supporting the conclusions of this article will be made available by the authors, without undue reservation.
