# Corrigendum: Immune Response in Myocardial Injury: *In Situ* Hybridization and Immunohistochemistry Techniques for SARS-CoV-2 Detection in COVID-19 Autopsies

**DOI:** 10.3389/fmolb.2022.887178

**Published:** 2022-03-31

**Authors:** Pek Yoon Chong, Jabed Iqbal, Joe Yeong, Tar Choon Aw, Kian Sing Chan, Paul Chui

**Affiliations:** ^1^ Department of Pathology, Sengkang General Hospital, Singapore, Singapore; ^2^ Department of Anatomical Pathology, Singapore General Hospital, Singapore, Singapore; ^3^ Institute of Molecular and Cell Biology, A-STAR, Singapore, Singapore; ^4^ Department of Molecular Pathology, Singapore General Hospital, Singapore, Singapore; ^5^ Health Science Authority, Singapore, Singapore

**Keywords:** PCR, autopsy, multiplex, serology, COVID-19

In the original article, we neglected to include the **Funder** “SingHealth Duke-NUS Pathology Academic Clinical Program (ACP).”

The corrected **Funding** statement appears below:

“This publication was funded by SingHealth Duke-NUS Pathology Academic Clinical Program (ACP).”

The authors apologize for this error and state that this does not change the scientific conclusions of the article in any way. The original article has been updated.

