# Supplementary material for: Immune Response in Myocardial Injury: In Situ Hybridization and Immunohistochemistry Techniques for SARS-CoV-2 Detection in COVID-19 Autopsies
Source: Front Mol Biosci. 2021 Oct 26;8:658932. doi: 10.3389/fmolb.2021.658932 (PMC8576174; doi:10.3389/fmolb.2021.658932)
Supplement: Supplementary file 1 [file Table1.DOCX]

Supplementary Table 1. Details of antibodies for multiplex IHC/IF

| **Antibody** | **Clone** | **Source** |
| --- | --- | --- |
| ACE2 | Abcam | ab108252 |
| CD3 | Dako | DKO.A045201 |
| CD38 | Leica Biosystems | CD38-290-L-CE |
| CD4 | NCL-L-CD4-368 | 4B12 |
| CD68 | Dako | DKO.M087601 |
| CD8 | Leica Biosystems | CD8-4B11-L-CE |
|  |  |  |
| Collagen I | Abcam | ab34710 |
| Collagen III | Abcam | ab7778 |
| GATA3 | Ventana | 760-4897 |
| GM-CSF | Novus Biologicals | NBP2-46364 |
| IFN-gamma | Santa Cruz | sc74108 |
| IL-1b | CST | #12242 |
| Ki67 | Dako | DKO.M724001 |
| SARS-CoV-2 NP | Novus Biologicals | NB100-56576 |
| SARS-CoV-2 NP | BioVision | A2061 |
| TMPRSS2 | Abcam | ab92323 |
| VEGF | Dako | M7273 |

Supplementary table 2: Microscopic cardiac findings in four non-COVID patients during the same period.

| Case no (age in y) | Coronary arteries | Myocardium | Cardiac vessels | Endocardium | Pericardium |
| --- | --- | --- | --- | --- | --- |
| 1 (40) | 80% occlusion; atherosclerosis; lymphocytic infiltrate | No inflammation | No inflammation | No inflammation | No inflammation |
| 2 (39) | 90% occlusion; fresh thrombus; no lymphocytic infiltrate | No inflammation; old infarct | No inflammation | No inflammation | No inflammation |
| 3 (51) | 80% occlusion; mild lymphocytic infiltrate | Nil | No inflammation | No inflammation | No inflammation |
| 4 (29) | 75% occlusion | Nil | No inflammation | No inflammation | No inflammation |
